# Supplementary material for: Limits on determining the skill of North Atlantic Ocean decadal predictions
Source: Nat Commun. 2018 Apr 27;9:1694. doi: 10.1038/s41467-018-04043-9 (PMC5923258; doi:10.1038/s41467-018-04043-9)
Supplement: Supplementary file 1 — Supplementary Information [file 41467_2018_4043_MOESM1_ESM.pdf]

Supplementary Information for "Limits on determining the skill of North Atlantic Ocean decadal predictions"

Matthew B. Menary[1], Leon Hermanson[1]

[1]Met Office Hadley Centre, Met Office, United Kingdom
March 14, 2018

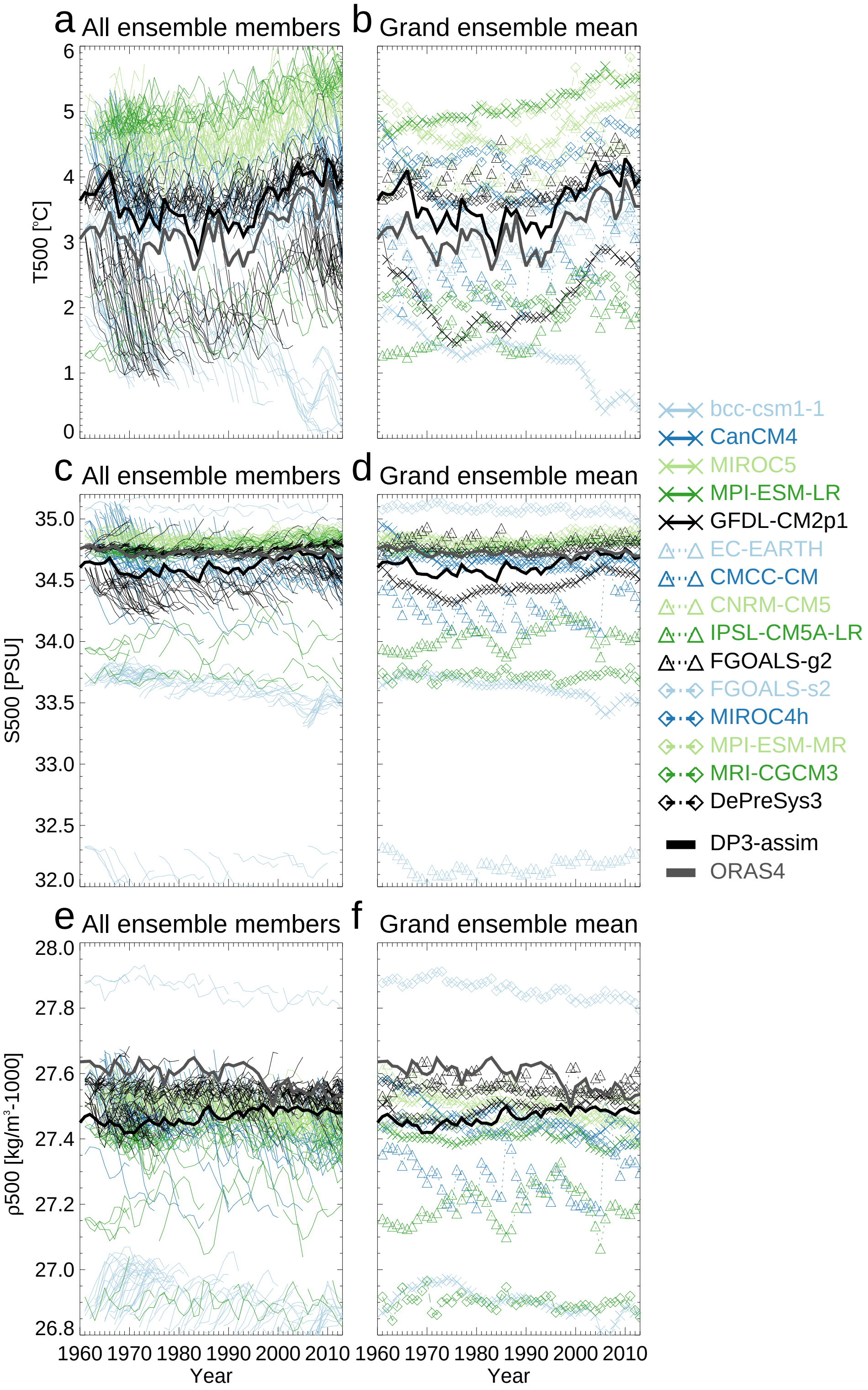

Supplementary Figure 1: **Uncorrected raw data.** Time series of volume averaged temperature ($T500$, a/b), salinity ($S500$, c/d), and density ($\rho500$, e/f) in the Labrador Sea (45–60°W, 55–65°N) top 500m in reanalyses and hindcast systems. The left column (a/c/e) shows all ensemble members and start dates. The right column (b/d/f) shows the mean over all ensemble members and lead times valid at the given year, but prior to any bias correction, which is applied in Figure 1 of the main manuscript.

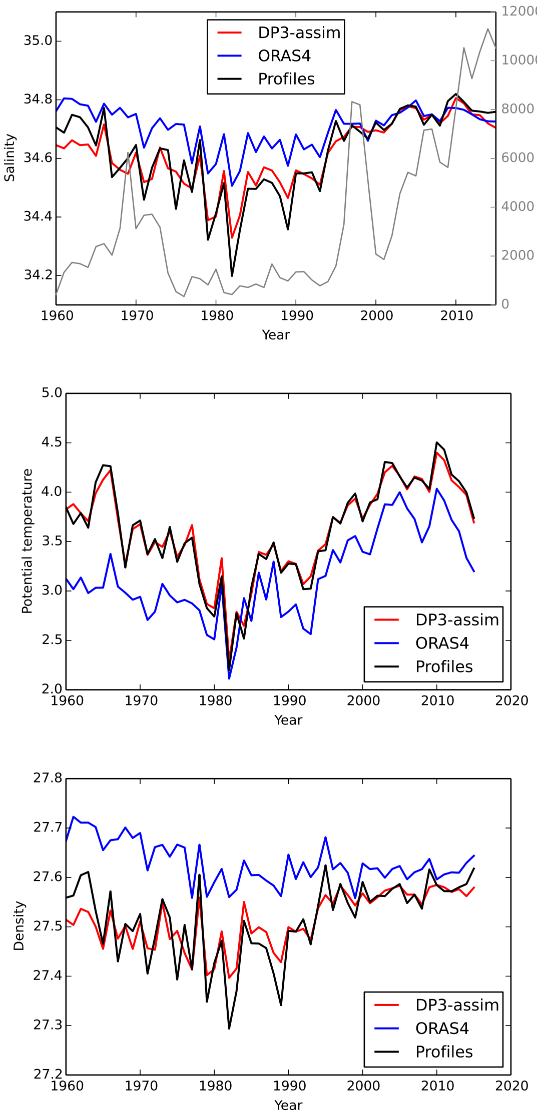

Supplementary Figure 2: **Comparison of reanalyses and instrumental profiles.** Annual mean salinity (top), temperature (middle), and density (bottom) for the top 500m of observed profiles within the Labrador Sea box (black) and ORAS4 (blue) and DP3-assim (red) subsampled at the same times and locations as the observations where there are joint temperature and salinity observations. Note that these annual means may be biased by observations not taking place uniformly in time or space. Even if there is only one profile in a year, then the mean of that profile will represent that year. The number of observations comprising each annual mean is shown on the grey line and right hand axis (top). This figure shows that both analyses capture the trends and interannual variability of the observed salinity changes. It appears that ORAS4 has a salty bias before the 1990s, but as the observations may be biased to a certain time of year and certain locations, this does not imply that this is a problem for the whole Labrador Sea all year round. Note that ORAS4 is regridded to the DP3-assim grid prior to masking, which may result in additional uncertainties. The profiles cannot be considered the absolute truth as they have sampling errors when gridded so in reality the actual salinity of the Labrador Sea may have been different. In addition, we show in the main manuscript that it is the interannual variability, which is captured in both models, that is key for understanding the drivers of density, not the mean state.

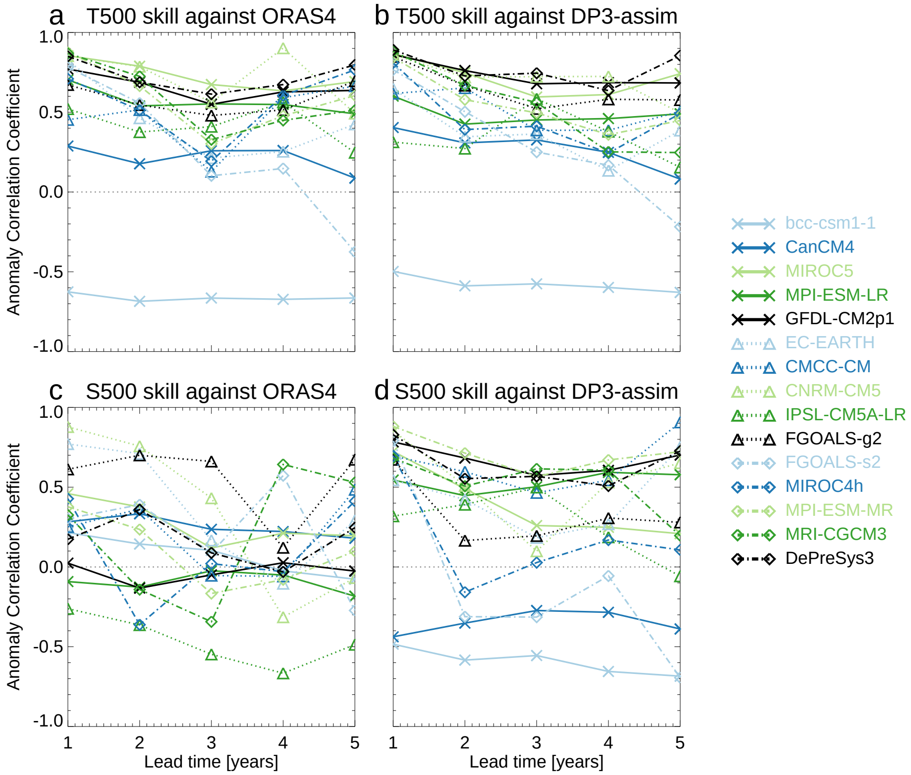

Supplementary Figure 3: **Temperature and salinity correlation skill.** Correlation skill in Labrador Sea top 500m temperature ($T500$) between hindcast systems and the reanalyses ORAS4 (a) and DP3-assim (b) as a function of lead time. c/d as a/b but for top 500m salinity ($S500$). A lead-time dependent bias-correction (assessed against ORAS4 and DP3-assim separately) is applied to the hindcasts before calculating the skill.

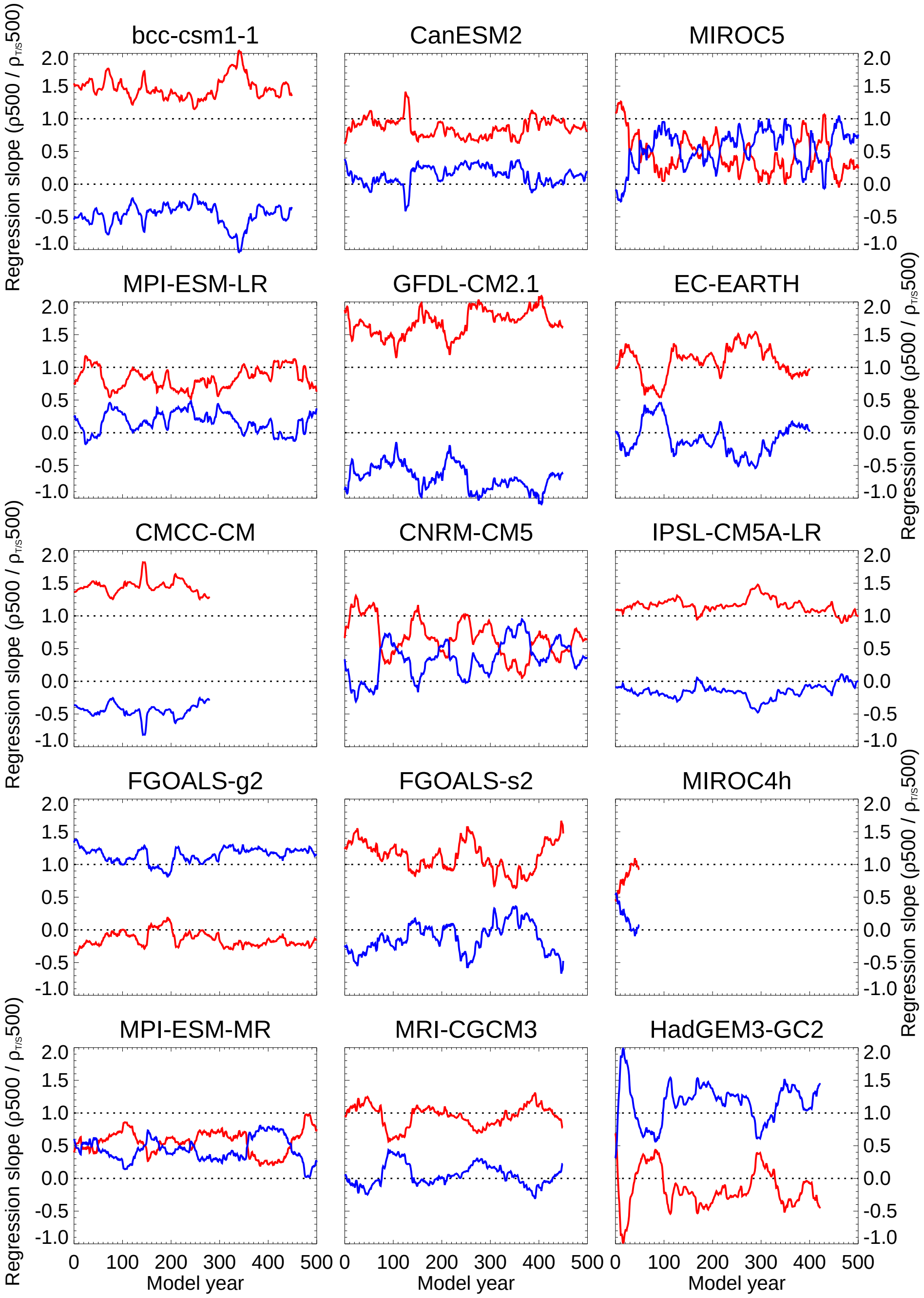

Supplementary Figure 4: **Stationarity of density driver in control simulations.** Regression slopes between $\rho 500$ and $\rho_T 500$ (blue) or $\rho 500$ and $\rho_S 500$ (red) in the control simulations using a 50 year running window.

References

[1] Yashayaev, I. & Loder, J. W. Recurrent replenishment of labrador sea water and associated decadal-scale variability. *Journal of Geophysical Research: Oceans* (2016).

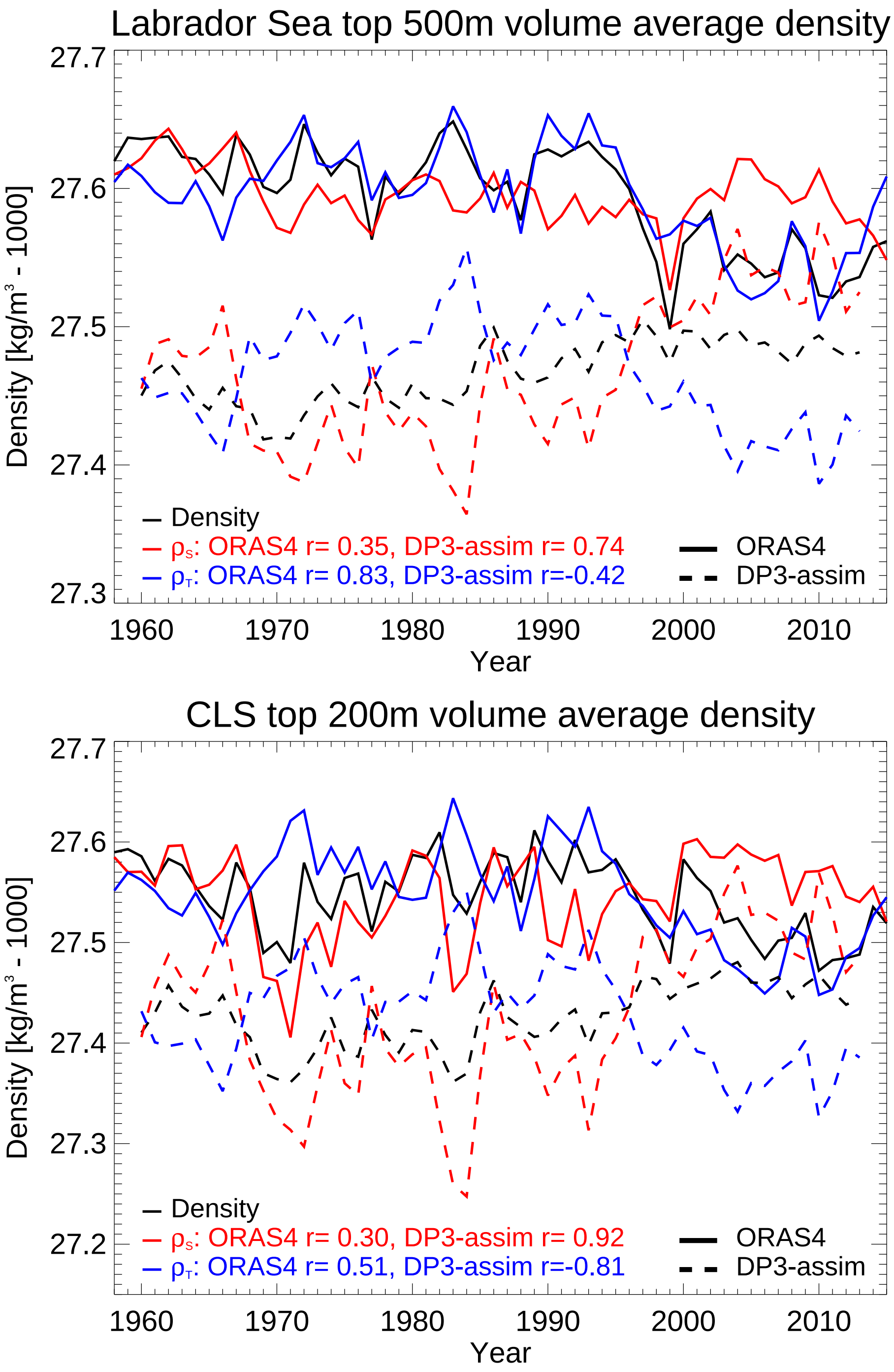

Supplementary Figure 5: **Sensitivity of density-driver to Labrador Sea definition.** Density (black) and its components (temperature, blue; salinity, red) in ORAS4 (solid) and DP3-assim (dashed) for the original region top 500m and the Central Labrador Sea (CLS, after ref [1]) top 200m. Correlations (r) between between full density and density due to temperature ($\rho_T 500$), and between full density and density due to temperature ($\rho_S 500$) are inlaid. In both regions, DP3-assim shows a high correlation between full density and $\rho_S 500$. In addition, in both regions, ORAS4 shows a preference for instead $\rho_T 500$, although the correlation is slightly weakened when using the smaller region.
